# An Approach for Prioritizing “Down-the-Drain” Chemicals Used in the Household

**DOI:** 10.3390/ijerph120201351

**Published:** 2015-01-26

**Authors:** Marina Rotsidou, Mark D. Scrimshaw

**Affiliations:** Institute of Environment, Health and Societies, Brunel University London, Uxbridge UB8 3PH, UK; E-Mail: m_rotsidou@hotmail.com

**Keywords:** emerging, contaminant, environment, water, quality, domestic, discharge

## Abstract

Many chemicals are present in cleaning and personal care products, which after use are washed down the drain and find their way into water bodies, where they may impact the environment. This study surveyed individuals to determine what products were used most in the home, in an attempt to prioritize which compounds may be of most concern. The survey resulted in the identification of 14 categories of products consisting of 315 specific brands. The survey estimated that individuals each discharge almost 33 L of products per year down the drain. Dishwashing liquids and hand wash gels, which accounted for 40% of this volume, were selected for identification of specific ingredients. Ingredients were classified as surfactants, preservatives, fragrances or miscellaneous, with hand wash gels having a wider range of ingredients than dishwashing liquids. A review of the literature suggested that preservatives, which are designed to be toxic, and fragrances, where data on toxicity are limited, should be prioritized. The approach undertaken has successfully estimated use and provisionally identified some classes of chemicals which may be of most concern when used in cleaning and personal care products.

## 1. Introduction

Products used in the home comprise a diverse range of leave-on and rinse-off formulations used for general hygiene and cleaning purposes, as well as for personal hygiene and cosmetic reasons [1]. Although cleaning and personal care products (PCP) are consumed in higher volumes than other categories of chemicals, such as pharmaceuticals, little is currently known regarding their effects on the aquatic environment, related potential toxicity or environmental concentrations [2,3]. The primary pathway for household chemicals to the aquatic environment is mediated by wastewater treatment works, which act as a barrier between the sewer and aquatic environment. However, although effective removal of some chemicals may occur [4], others pass through the treatment processes to pose a possible risk to the environment and human health [1,5,6].

A range of chemicals related to PCP have been reviewed for occurrence and toxicity, highlighting areas for concern [2], and evidence shows that some of these chemicals and/or their biodegradation products found in sewage effluent and subsequently surface waters have been shown to be harmful to aquatic organisms [1,7]. The use of a myriad of cleaning and PCP on a daily basis and the introduction of new chemicals every year can therefore possibly pose a risk to aquatic organisms.

This aim of this work is to develop an approach which will help identify chemicals disposed of “down the drain” by households. It is based on obtaining information on the products used, and determining what chemicals they contain. This was developed by gathering information on the use of products and the development of a methodology which prioritized the chemicals based on their use and potential to effect the aquatic environment.

## 2. Experimental Section

### 2.1. Design of the Questionnaire

The study focused on developing an inventory of consumer products which were most frequently used in UK households. A survey was carried out from 2 June to 3 July 2014 and the data were collected through a questionnaire. All subjects gave their informed consent for inclusion before they participated in the study, which followed the guidelines provided for research within the Institute for the Environment at Brunel University. The questionnaire was distributed to 150 postgraduate students and staff of the Institute for the Environmental at Brunel University, resulting in a sample of working age, mixed sex individuals. No personal details were collected from participants, who listed up to ten products they most frequently used in the kitchen and bathroom. The respondents were asked to provide full product descriptions, the frequency of use (daily, weekly or monthly) and to estimate the quantity used. Two examples of how the products should be described were given on the questionnaire (see supplementary file).

### 2.2. Estimation of Use from the Questionnaire Data

The amount of each product used was expressed as the average use (in L) per respondent per year, based on Equation (1). As the questionnaire asked users to estimate volumes (or masses used), all calculations assumed that 1 mL≡1 g. In the questionnaire, quantities were expressed as 0–10, 10–100 or >100 mL (or g) and in the calculation, values of 3, 30 or 300 (mL) were substituted into “quantity used” for each of these responses respectively. The objective of the calculation was to give an estimate for the volume used, which would allow for the determination of which products were used more than others. For frequency of use, values of 30 (daily), 4 (weekly) and 1 for monthly use were used. The calculation then averaged use amongst all respondents and multiplied that average by 12 to give an estimate of average annual use per product per respondent.

(1) $$\left[\frac{\left(quantity\;used\right)\;\times \left(frequency\;of\;use\right)\times \left(number\;of\;users\right)}{\left(number\;of\;respondents\right)\times 1000}\right]\times 12$$

## 3. Results

The survey resulted in the return of 52 completed questionnaires, the analysis of which resulted in the identification of 315 different products used in the household by respondents. The initial challenge was how to begin assessing information about such a large number of individually branded products, and to facilitate dealing with this issue, the products were allocated into categories according to the details provided by the respondents. In all, 14 categories to which products could be allocated were identified, and these fell into either cleaning products or PCP (Table 1). Some of these categories, such as “household cleaners” were relatively broad, whilst others, such as “toothpastes” were quite specific. In some cases the number of users for each category exceeded the number of respondents, as more than one product from each category were used by some of the individual respondents.

The responses provide an overview of product use and also data on how the respondents may perceive the amount used. The questionnaire asked for information on the 10 products most frequently used, and for the amount used to then be estimated. Overall, 14 product categories were identified, with use ranging from 0.08 to 7.98 L per person per year (L·per^−1^·yr^−1^) with a total of 32.78 L·per^−1^·yr^−1^ used and potentially discharged to the drain.

**Table 1:** The categories of cleaning products and PCP identified and the number of times respondents identified using products in each category in brackets.

Cleaning Products		PCP	
1. Household cleaners (bleaches, disinfectants, lime scale removers, kitchen cleaners *etc.*)	(72)	1. Toothpastes	(48)
2. Dishwashing liquids	(51)	2. Shampoos	(47)
3. Laundry products (washing powders, washing tablets, laundry gels, fabric conditioners)	(46)	3. Body-wash gels	(39)
4. Dishwasher detergents	(10)	4. Hand wash gels	(34)
--		5. Hair conditioners	(28)
		6. Deodorants	(21)
		7. Face wash products	(16)
		8. Face creams	(11)
		9. Soap bars	(10)
		10. Shaving products (gels/foams after shaves and balms)	(8)

### 3.1. Prioritization of Product Categories

**Figure 1:**
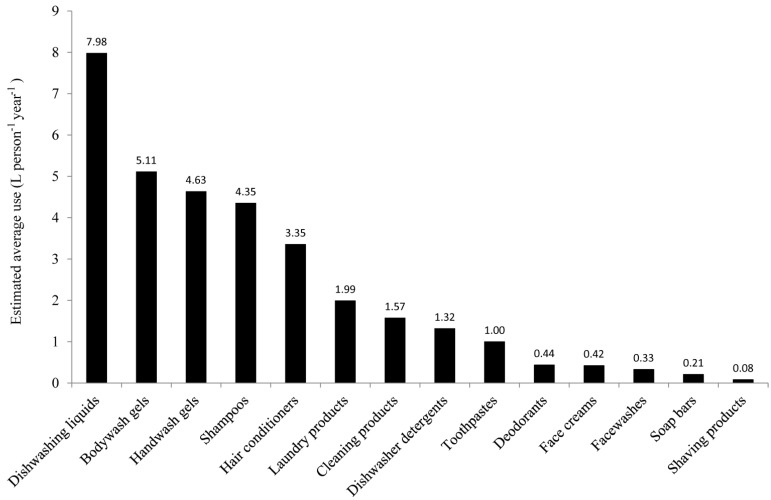
Prioritization of product categories based on the estimated average use (L) per person per year. Detail showing the calculation for the dishwashing liquids is shown in Table 2.

The prioritization of the product categories was undertaken using Equation (1), which gave the average annual use per person (Figure 1). The data in Figure 1 are based on estimates of use, and may therefore not reflect with a high degree of accuracy actual volumes used. However, unless there was a consistent under or overestimate for any product by most respondents, the data should reflect what are the highest to least used products. The outcome demonstrated that dishwashing liquids, used to hand wash cutlery and crockery in the sink, were the products estimated to be used most, followed by a range of PCP used for cleaning the body, hands and hair. Next in sequence were three more cleaning products (for laundry, more general household cleaning products and dishwasher detergents), followed by a range of PCP, from toothpastes to shaving products.

Having prioritized the product categories by the volume used, the next logical step in development of the methodology was to investigate further the brands that respondents used within each category. For the dishwashing liquids this breakdown is shown in Table 2, with Fairy being the most popular brand (78% of users). However, with seven different types of this brand, which from the product descriptions were apparently based on fragrance combinations, the complexity of the task was becoming apparent. Overall, Fairy Original and Fairy Lemon were the dishwashing liquids that dominated this category. The majority of those using these products (73%) estimated use as between 10 and 100 mL a day.

**Table 2:** The 14 different dishwashing liquids as reported by the 51 respondents who used these products, in order of decreasing average use. The frequency was identified as “daily” by all respondents, so columns for weekly and monthly are omitted.

Brand	Full Description	Users	Frequency	Estimate of Use (mL)			Average Use
			Daily	0–10	10–100	>100	L·per^−1^·yr^−1^
Fairy	Original	17	17	4	13		2.78
Fairy	Lemon	13	13	1	12		2.51
Fairy	Apple & Orchard	4	4		4		0.83
Fairy	Platinum	2	2		2		0.42
Fairy	Chamomile & vitE	1	1		1		0.21
Fairy	Orange & Lemongrass	1	1		1		0.21
Fairy	Pomegranate & Honey suckle	1	1		1		0.21
Tesco	Lime & Lemongrass antibacterial	1	1		1		0.21
W5	Lemon	1	1		1		0.21
Sainsbury’s	Original	1	1		1		0.21
Ecover	Lemon & Aloe Vera	6	6	6			0.12
Easy	With a hint of Verbena	1	1	1			0.02
Fairy	Platinum Lemon	1	1	1			0.02
Magnum	Original	1	1	1			0.02
Total		51	51	14	37		7.98

The product category with second highest use, body-wash gels, displayed a much more complex mix of brands, although the Dove brand appeared to dominate, with average use of these products by the 52 respondents being 1.54 L·per^−1^·yr^−1^, or 30% of the body-wash used. In manner similar to the dishwashing liquids, the use of terms describing fragrance or flavor was apparent in many of the names of products, such as “Pink Grapefruit” or “Power Fruits”. It was also apparent that people who use these products do so daily, and the majority of users (24) estimated use to be between 10 and 100 mL per day (Table 3).

A full breakdown of brands and individual types of product for the other categories identified in Table 1 and Figure 1 was also undertaken and the outcomes are summarized in Table 4. A full breakdown of each of these categories is provided in Tables S1–S12. The category of cleaning products is the most diverse, covering products used in kitchens, bathrooms and toilets, for general cleaning and specific tasks such as disinfection and lime scale removal. They comprise a wide range of forms, liquids, gels, sprays and powders. Although these products were identified most commonly by respondents, their less frequent, predominantly weekly use, in combination with estimated volumes, resulted in the lower overall usage reported in Figure 1.

### 3.2. Identification and Classification of Chemicals in the Products

After the prioritization of the 14 product categories, the next step was to identify the chemicals used in the products. With a total of 315 products over the 14 categories, this task needed to be made manageable to meet the objective of the work. The two categories of products used most, dishwasher liquids and body-wash, which accounted for 40% of the total volume of 32.78 L·per^−1^·yr^−1^ (Figure 1), were selected to determine if chemicals used as ingredients could be identified, evaluated and prioritized.

**Table 3:** The 31 different body-washes as reported by the 39 respondents who used these products, in order of decreasing average use.

Brand	Full Description	Users	Frequency	Estimate of Use (mL)			Average Use
			Daily	0–10	10–100	>100	L·per^−1^·yr^−1^
Dove	Deep Moisture BSG	4	4	2	2		0.46
Dove	Men + Care (original) BW	2	2		2		0.42
Original source	Mint Shower (for men) BSG	2	2	1	1		0.23
Dove	Deeply nourishing BW	2	2	1	1		0.23
Sanex	0% for dry skin SG	2	2	1	1		0.23
Dove	Silk Glow BC	1	1		1		0.21
Dove	Supreme Body Silk Body Cream	1	1		1		0.21
Body Shop	Pink Grapefruit SG	1	1		1		0.21
Funky Farm	Baylis & Harding BSG	1	1		1		0.21
Garnier	Pure active fruit BSG	1	1		1		0.21
L’Occitane	Rose BSG	1	1		1		0.21
Nivea	Power Fruits Refreshing BSG	1	1		1		0.21
Nivea	Sensitive (Chamomile extracts) BSG	1	1		1		0.21
Palmolive	Aroma Therapy (anti-stress) BSG	1	1		1		0.21
Rock Face	For men BSG	1	1		1		0.21
Sanex	Hypo-allergenic BSG	1	1		1		0.21
Imperial Leather	Aqua Therapy Bath Soak	1	1		1		0.21
Cien	Foam Bath Moisturising BW	1	1		1		0.21
Johnson’s	Baby Bedtime Bath BW	1	1		1		0.21
Radox	Moisturise (shower cream) BW	1	1		1		0.21
Sanctuary	Spa BW	1	1		1		0.21
Palmolive	Milk and Honey SM	2	2	1	1		0.04
Palmolive	Nourishing Delight Bath Milk	1	1	1			0.02
Dove	Diamond Touch BSG	1	1	1			0.02
Nivea	Original Care (for men) BSG	1	1	1			0.02
Radox	Key Lime & Peppermint BSG	1	1	1			0.02
Soap & Glory	Clean On Me Creamy Clarifying BSG	1	1	1			0.02
Superdrug	Sweet Sixties BSG	1	1	1			0.02
Treacle Moon	Raspberry Kiss BSG	1	1	1			0.02
Imperial Leather	Jasmine & Cotton Milk BW	1	1	1			0.02
Victoria’s Secret	Red Plum & Freesia BW	1	1	1			0.02
Total		39	39	15	24		5.11

BC Body Cream; BSG Body Shower Gel; BW Body-wash; SG Shower Gel; SM Shower Milk.

**Table 4:** A summary of the 12 remaining product categories and the number of different products within each category. For full details see Tables S1–S12.

Product Category	Number of Products
Hand wash gels	26
Shampoos	37
Hair conditioners	26
Laundry products	36
Cleaning products	47
Dishwasher detergents	8
Toothpastes	31
Deodorants	19
Face creams	11
Face washes	16
Soap bars	6
Shaving products	7

A total of 116 different ingredients were identified from these two most-used categories. To facilitate the prioritization exercise, these were classified by functionality in the formulation. This resulted in the derivation of three clear classes, surfactants, preservatives and fragrances. Compounds which did not readily fit into these classes were placed in a “miscellaneous” class. Emulsifiers were assessed with the surfactants, given their similar functionality, and anti-oxidants were included in the class of preservatives. This step was, however, challenging for many chemicals, since their functionality did not clearly relate to one class and many are reported to have more than one role in product formulations. For example, benzyl alcohol is used as both a preservative and a fragrance, whereas benzophenone has functionality as a UV filter and fragrance [8,9,10]. The miscellaneous class included thickeners (such as xanthan gum), chelating agents (e.g., EDTA), UV stabilizers (e.g., benzotriazolyl dodecyl p-cresol), pH regulators (e.g., lactic acid), artificial colors (e.g., CI 17200) and inorganic salts.

#### 3.2.1. Chemicals in Dishwashing Products

Identification of the ingredients was obtained from the labels of ten out of the 14 dishwashing liquids identified in Table 2, since only these were readily available. The chemicals listed for each dishwashing liquid included those in the seven Fairy dishwashing liquids along with the Ecover, Tesco and Sainsbury’s products (Table 5). The number of ingredients in this product category ranged from five for the Fairy “Original” and “Lemon” to 12 for the Sainsbury’s and Tesco dishwashing liquids.

Apart from water (which was estimated to be around 40%–50% by volume), the key ingredients in the dishwashing products were anionic and non-ionic surfactants, with only two also containing amphoteric surfactants (Table 5). Nine out of these ten dishwashing liquids gave the percentage of the surfactants used in the product from 5% to 30% for anionic surfactants, <5% to 15% for non-ionic surfactants and 5% to 15% amphoteric surfactants. Only the Ecover product was labelled differently, with more detailed description containing the commercial names of two surfactants, namely sodium lauryl ether sulfate and alkyl polyglycoside C10-16.

**Table 5:** The ingredients identified in ten dishwashing liquids, allocated to classes.

Product	Ingredients
Fairy Original	*Surfactants*: 15%–30% anionic surfactants, 5%–15% non-ionic surfactants
	*Preservatives*: Methylisothiazolinone, phenoxyethanol
	*Fragrances*: “perfume”
Fairy Lemon	*Surfactants*: 15%–30% anionic surfactants, 5%–15% non-ionic surfactants
	*Preservatives*: Methylisothiazolinone, phenoxyethanol
	*Fragrances*: “perfume”
Fairy Platinum	*Surfactants*: 15%–30% anionic surfactants, 5%–15% non-ionic surfactants
	*Preservatives*: Methylisothiazolinone, phenoxyethanol
	*Fragrances*: Butylphenyl methylpropional, hexyl cinnamal, limonene, “perfume”
Fairy Platinum Lemon	*Surfactants*: 15%–30% anionic surfactants, 5%–15% non-ionic surfactants
	*Preservatives*: Methylisothiazolinone, phenoxyethanol
	*Fragrances*: Hexyl cinnamal, limonene, “perfume”
Fairy Apple and Orchard	*Surfactants*: 5%–15% anionic surfactants, <5% non-ionic surfactants
	*Preservatives*: Methylisothiazolinone, phenoxyethanol
	*Fragrances*: Geraniol, linalool, limonene, “perfumes”
Fairy Chamomile and vitE	*Surfactants*: 5%–15% anionic surfactants, <5% non-ionic surfactant
	*Preservatives*: Benzisothiazolinone, phenoxyethanol
	*Fragrances*: Geraniol, limonene, “perfume”
Fairy Pomegranate and Honeysuckle	*Surfactants*: 5%–15% anionic surfactants, <5% non-ionic surfactants
	*Preservatives*: Methylisothiazolinone, phenoxyethanol
	*Fragrances*: Butylphenyl methylpropional, hexyl cinnamal, linalool, “perfume”
Ecover Lemon and Aloe Vera	*Surfactants*: Alkyl poly glycoside C10-16, sodium lauryl ether sulphate
	*Preservatives*: Citric acid, 2-bromo-2-nitropropane-1,3-diol
	*Fragrances*: Aloe Barbadensis extract, citral, limonene, “perfume”
Tesco Lime and Lemongrass Antibacterial	*Surfactants*: 15%–30% anionic surfactants, 5%–15% amphoteric surfactants, >5% non-ionic surfactants
	*Preservatives*: 2-bromo-2-nitropropane-1,3-diol (0.1 g per 100 g), benzisothiazolinone, dimethylol glycol (0.08 g per 100 g), methylchloroisothiazolinone (0.00105 g per 100 g), methylisothiazolinone (0.00035 g per 100 g)
	*Fragrances*: Limonene, linalool, “perfume”
	*Miscellaneous*: Laurylamine dipropylenediamine
Sainsbury’s Original	*Surfactants:* 15%–30% anionic surfactant, <5% non-Ionic surfactant, 5%–15% amphoteric surfactant
	*Preservatives*: Benzisothiazolinone, dimethylol glycol, iodopropynyl butylcarbamate
	*Fragrances*: Citral, limonene, “perfume”
	*Miscellaneous*: Laurylamine dipropylenediamine, protein hydrolysate, sodium chloride

Regarding the preservatives, eight different ingredients of this class were identified in the dishwashing liquids, with methylisothiazolinone and phenoxyethanol being the most frequently observed chemicals in the Fairy dishwashing liquids. Fairy “Chamomile and vitE” included the preservative benzisothiazolinone instead of methylisothiazolinone, which was also contained in the label list of Tesco’s and Sainsbury’s dishwashing liquids. Citric acid and 2-bromo-2-nitropropane-1,3-diol were identified in Ecover, with the latter being also identified in Tesco’s dishwashing liquid. The Tesco’s product was labelled differently, with information also giving the quantity of the preservatives.

The class of fragrances included five different chemicals, of which limonene was the most frequently used and the term “perfume” was listed in all the dishwashing products.

#### 3.2.2. Chemicals in Body-Wash Products

From the second most used category, body-washes, information on the ingredients was readily available for only 12 out of 31 products that were reported as used in the survey. In comparison to dishwashing liquids, a wider range of brands was identified, while also a higher number of ingredients was observed, which ranged from 11 for Sanex “0% for Dry Skin” shower gel to 33 for Dove “Deeply Nourishing Body-wash” (Table 6).

The body-washes were, as dishwashing liquids, also surfactant-based, often giving the name of the specific surfactant. However unlike dishwashing liquids, the percentage amount of this ingredient was not provided in any product. More specifically, body-washes included a number of anionic (e.g., sodium laureth sulphate and sodium C12-13 pareth sulphate) and non-ionic (e.g., cocamide DEA, cocamide MEA and coco-glycoside) surfactants. Amphoteric surfactants were also present, the most prominent being cocamidopropyl betaine. The ingredient list of body-washes, in contrast to dishwashing liquids, also included emulsifiers (e.g., PEG-7 glyceryl cocoate, PEG-40 hydrogenated castor oil and poloxamer 124), which are listed with surfactants in Table 6.

There were 16 different preservatives identified, twice the number found in dishwashing liquids. However, both categories of products had some of this class of chemicals in common, such as citric acid, methylchloroisothiazolinone and methylisothiazolinone. As preservatives in the body-washes, citric acid and sodium benzoate were the most frequently identified ingredients (nine times each), and two forms of parabens (methyl- and propyl- paraben) were identified in one body-wash. The class of preservatives also included antioxidants, which were not present in dishwashing liquids. The antioxidants identified were BHT, retinyl palmitate, sodium ascorbyl phosphate, tocopherol and tocopheryl acetate.

A wider range of fragrances were also present in body-washes, with 11 different ingredients identified in this class. The four most frequently observed were limonene, linalool, butylphenyl methylpropional and hexyl cinnamal, which were identified seven, six, five and four times, respectively. All these four chemicals were also used as fragrances in dishwashing liquids. Additionally, benzophenone-4 was presentd in two body-washes, whereas the remaining chemicals, alpha-isomethyl ionone, amyl cinnamal, benzyl benzoate, hydroxyisohexyl 3-cyclohexene carboxaldehyde and coumarin, were identified just once. As in dishwashing liquids, the term “perfume” was included as an ingredient in all the body-washes.

**Table 6:** The ingredients identified in 12 body-wash products, allocated to classes.

Body-Wash	Ingredients
Dove Men + Care Original BW	*Surfactants*: Cocamide MEA, sodium laureth sulfate
	*Preservatives*: BHT, DMDM hydantoin
	*Fragrances*: Alpha-isomethyl ionone, butylphenyl methylpropional, coumarin, hexyl cinnamal, limonene, linalool, “perfume”
	*Miscellaneous*: Acrylates copolymer, CI 17200, CI 19140, CI 42090, petrolatum, sodium chloride, sodium hydroxide, tetrasodium EDTA
	*Preservatives*: BHT, DMDM hydantoin
Dove Deeply Nourishing BW	*Surfactants*: Cocamidopropyl betaine, sodium cocoylglycinate, sodium hydroxypropyl starch phosphate, sodium isethionate, sodium laureth sulphate, sodium lauroyl isethionate, sodium palm kernelate, sodium palmitate, sodium stearate, stearic acid
	*Preservatives*: Benzyl alcohol, BHT, citric acid, DMDM hydantoin, methylisothiazolinone, sodium benzoate
	*Fragrances*: Butylphenyl methylpropional, citronellol, hexyl cinnamal, limonene, linalool, “perfume”
	*Miscellaneous*: Alumina, CI 77891, glycerin, guar hydroxypropyltrimonium chloride, Helianthus annuus hybrid oil, hydrogenated soybean oil, lauric acid, sodium chloride, tetrasodium EDTA, tetrasodium etidronate, zinc oxide
Nivea Power Fruits Refreshing SG	*Surfactants*: Cocamidopropyl betaine, PEG-7 glyceryl cocoate, PEG-40 hydrogenated castor oil, PEG-200 hydrogenated glyceryl palmate, sodium laureth sulphate
	*Preservatives*: Citric acid, sodium ascorbyl phosphate, sodium benzoate
	*Fragrances*: Benzophenone-4, butylphenyl methylpropional, limonene, linalool, “perfume”
	*Miscellaneous*: CI 16035, glycerin, glycerylglucoside, Helianthus annuus seed oil, polyquaternium-7, sodium chloride, Vacciniummacrocarpon fruit juice
Original Source Mint Shower for men SG	*Surfactants*: Cocamidopropyl betaine, lauryl glucoside, sodium laureth sulphate
	*Preservatives*: BHT, sodium benzoate
	*Fragrances*: Limonene
	*Miscellaneous*: Benzotriazolyl dodecyl p-cresol, CI 19140, CI 42090, lactic acid, Melaleucaalternifolia (tea tree) leaf oil, Menthaarvensis (peppermint) leaf oil, sodium chloride, styrene/acrylates copolymer, tetrasodium glutamate diacetate
Sanex Hypo-Allergic SG	*Surfactants*: Cocamidopropyl betaine, coco-glucoside, sodium laurethsulfate
	*Preservatives*: Caprylyl glycol, sodium benzoate
	*Fragrances*: “perfume”
	*Miscellaneous*: Glycerin, glyceryloleate, lactic acid, polyquaternium-7, sodium chloride, sodium lactate, zinc sulphate
Sanex 0% for Dry Skin SG	*Surfactants*: Cocamidopropyl betaine, coco-glucoside, sodium laureth sulphate
	*Preservatives*: Caprylyl glycol, sodium benzoate
	*Fragrances*: “perfume”
	*Miscellaneous*: Glycerin, glyceryloleate, lactic acid, sodium chloride, sodium lactate
Funky Farm Baylis and Harding SG	*Surfactants*: Cocamide DEA, cocamidopropyl betaine, coco-glucoside, sodium laureth sulphate
	*Preservatives*: Benzyl alcohol, methylchloroisothiazolinone, methylisothiazolinone
	*Fragrances*: Citric acid, “perfume”
	*Miscellaneous*: Magnesium chloride, magnesium nitrate, sodium chloride
Johnson’s Baby Bedtime Bath BW	*Surfactants*: Cocamidopropyl betaine, disodium lauroamphodiacetate, PEG-80 sorbitan laurate, PEG 150 Disterate, polysorbate 20, sodium laureth sulphate, sodium lauroamphoacetate
	*Preservatives*: Citric acid, sodium benzoate
	*Fragrances*: “perfume”
	*Miscellaneous*: Sodium chloride, sodium glycolate
Radox Moisturise SC	*Surfactants*: Cocamidopropyl betaine, PEG-3 distearate, sodium laureth sulphate
	*Preservatives*: Benzoic acid, citric acid, sodium benzoate
	*Fragrances*: Hexyl cinnamal, hydroxyisohexyl 3-cyclohexene carboxaldehyde, “perfume”
	*Miscellaneous*: Chamomilla recutita extract, dipropylene glycol, hydrolysed silk, lactis proteinum, lactose, polyquaternium-7, propylene glycol, Simmondsla chinensis oil, sorbitol, sodium chloride, sodium lactate, styrene/acrylates copolymer
Palmolive Milk and Honey SM	*Surfactants*: Cocamide MEA, cocamidopropyl betaine, laureth-4, poloxamer 124, sodium C12-13 pareth sulfate, sodium laureth sulfate
	*Preservatives*: Citric acid, retinyl palmitate, sodium benzoate, tocopherol
	*Fragrances*: Amyl cinnamal, benzyl benzoate, hexyl cinnamal, limonene, “perfume”
	*Miscellaneous*: Aloe barbadensis extract, CI 19140, CI 16255, glycerin, lactose, lactis serum proteinum, linoleic acid, mel, sodium chloride, tetrasodium EDTA
Treacle Moon Raspberry Kiss SG	*Surfactants*: Cocamide DEA, cocamidopropyl betaine, sodium laureth sulphate
	*Preservatives*: Benzyl alcohol, citric acid, methylchloroisothiazolinone, methylisothiazolinone, methylparaben, propylparaben
	*Fragrances*: Linalool, “perfume”
	*Miscellaneous*: CI 14700 (red 4), CI 17200 (red 33), CI 42090 (blue 1), glycerin, magnesium chloride, magnesium nitrate, polyquaternium-7, sodium chloride, styrene/acrylates copolymer, xanthan gum
Victoria’s Secret Red Plum and Freesia BW	*Surfactants*: Cocamide MEA, cocamidopropyl betaine, PEG 120 methyl glucose dioleate, PEG-150 disterate, sodium laureth -12 sulfate, sodium laureth sulfate, sodium lauryl sulfate
	*Preservatives*: Benzyl alcohol, citric acid, DMDM hydantoin, tocopheryl acetate, iodopropynyl butylcarbamate
	*Fragrances*: Benzophenone-4, butylphenyl methylpropional, limonene, linalool, “perfume”
	*Miscellaneous*: Aloe Barbadensis extract, CI 17200 (red 33), CI 16035 (red 40), sodium chloride

BC Body Cream; BSG Body Shower Gel; BW Body-wash; SG Shower Gel; SM Shower Milk.

## 4. Discussion

The questionnaire asked respondents about how much of a product was used and how frequently, however, for some products, clearly use is at a household, rather than an individual, level. No differentiation was made in this respect, as it would have added complexity, although it is a possible source of error. It also suggested categories of chemicals, which were added following feedback on a trial version, where respondents were unclear about what was included. A clear, simple, questionnaire was important in obtaining a good response. There are, however, indications that responses do reflect actual use of products.

**Figure 2:**
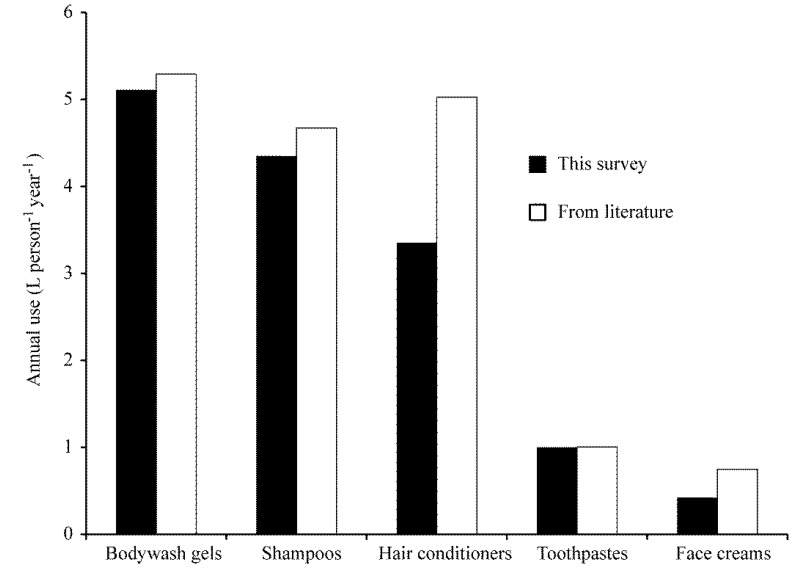
Comparison of annual use of categories in the survey with data available in the literature.

In the dishwashing liquids category, Fairy was the brand most frequently reported which is consistent with UK market research data, which show that Fairy dishwashing products dominate, with a 43% market share [11]. This trend was also reflected in the brands of dishwasher detergents, where Finish was the most popular brand, which also has a large (56%) market share in the UK [12,13]. The responses and subsequent calculation of annual use in L·per^−1^·yr^−1^ for a range of products used in the survey also agrees well with values reported in the literature [14,15,16,17] (Figure 2). These results demonstrate that the approach used to estimate use has produced data consistent with other research, which therefore gives a high degree of confidence that values derived are likely to be reasonable estimates of actual use.

### 4.1. The Environmental Significance of Surfactants in the Products

Surfactants constitute the key ingredients in dishwashing liquids and body-washes, with three types of surfactants identified in these two mostly used categories, namely anionic, non-ionic and amphoteric surfactants The occurrence and effects of surfactants have been studied extensively in comparison to preservatives and fragrances [8,18]. The linear alkyl sulphonates (LAS) are the most commercially important anionic surfactant globally, constituting 40% of the total sales of surfactants and more than 80% of those used in detergents in Europe [19]. After being discharged, LAS are removed by up to 99% in aerobic wastewater treatment processes [20], with further degradation in river water [21]. There is evidence that the risk posed by LAS to the aquatic environment is low, with the predicted environmental concentration (PEC) being below the predicted no-effect concentration (PNEC) for all the environmental compartments tested [19].

Other anionic surfactants, such as alcohol ethoxylates (AE) and alcohol ethoxysulfates (AES), are also readily degradable [21]. Their elimination rates in wastewater treatment are also high, indicating that there is little possibility of reaching the aquatic environment via sewage effluent and therefore they are expected to pose low aquatic risk [7,22]. The environmental risk assessment conducted for AE at 29 sites in Europe, Canada and the United States revealed that PEC/PNEC was well below 1, ranging between 0.049 and 0.094 [23], indicating that again, the risks posed to the aquatic environment by AE and AES are low.

Cocamidopropyl betaine is the commercial name of the amphoteric surfactant identified in many of the body-washes (Table 6). Cocamidopropyl betaine exhibits harmful/toxic effects to many aquatic test organisms in concentrations of 1–10 mg·L^−1^. However, in the aquatic risk assessment conducted by Gheorghe *et al.* (2013) for rivers in Romania [24], cocamidopropyl betaine was assessed to be safe for the aquatic environment with risk coefficients (PEC/PNEC) ranging from 0.036 to 0.38. Moreover, cocamidopropyl betaine is extensively degraded during wastewater treatment, is not bioaccumulative and not expected to cause long-term harmful effects in the local aquatic biota [25]. Overall, indications are that although surfactants constitute a significant volume of material discharged from households, they are not compounds of immediate concern.

### 4.2. The Environmental Significance of Preservatives in the Products

Chemicals used as preservatives are responsible for the inhibition of growth of bacteria in consumer products [26,27], and therefore are designed to have adverse effects on organisms. In this study, a wide range of preservatives have been identified in dishwashing liquids and body-washes. However, in comparison to detergents, there is less knowledge of the fate and occurrence of many of these compounds in the aquatic environment.

Preservatives which have the potential to present risks to the aquatic environment were identified, such as the two antimicrobial parabens compounds, methylparaben and propylparaben, which have been associated with weak estrogenic activity and low toxicity [28,29,30], and in mixtures with other estrogenic compounds can enhance the response [26]. Another example of a preservative with potential risk for aquatic life is 2-bromo-nitropropane-1,3-diol, which was identified in two dishwashing liquids Although not bioaccumulative, it is a preservative that has been classified as “very toxic to aquatic organisms” [25]. The anti-oxidant BHT, which was identified in three body-washes, is a further example of a preservative of potential concern, being a persistent, toxic and bioaccumulative compound, which has been previously detected in grey wastewater, wastewater and river water samples [29]. There was, therefore, some evidence that the preservatives present in products from households may pose a threat to the receiving waters following wastewater treatment. There is no indication on any of the products of how much preservative is present, which if available might allow for derivation of a PEC through modelling exercises.

### 4.3. The Environmental Significance of Fragrances in the Products

Fragrances are used ubiquitously in cleaning and PCP [31] and this is demonstrated in this survey, where five and 11 fragrances were identified in dishwashing liquids and body-washes respectively. In contrast to polycyclic-musks (PCMs) and nitromusks (not present in products identified in this survey), which have been studied thoroughly regarding their fate and effects on the aquatic environment, the potential aquatic impact of many fragrance compounds is largely unknown [8,32,33].

From the fragrances identified in this survey, limonene is an example of a fragrance with an indication of hazardous properties classified as “very dangerous to the aquatic environment”, with high acute toxicity to a range of aquatic species [8,34]. The most sensitive species to limonene was *Daphnia magna*, with a lowest reported acute toxicity of 0.4 mg·L^−1^ in a 48-h exposure [34]; however, no chronic exposure data, where effects are possible at lower concentrations, are available. The reported concentrations of limonene in surface waters are at least 250 times below the acute effect concentration; however, these data are more than 15 years old. The increasing pattern of use of fragrances in general [33] and the low dilution capacity of rivers in countries such as the UK [35], suggest that limonene may pose a risk to the aquatic environment.

Two other fragrances, coumarin and benzophenone, identified in body-washes, may also have negative impacts on aquatic organisms. Both these fragrances exhibit estrogenic activity, while benzophenone has also been classified as “very toxic to aquatic organisms”, with an acute EC_50_ to *D. magna* of 0.28 mgL^−1^ [8]. However, although environmental concentrations are likely to be well below this, with concentrations in both raw and treated drinking water ranging from 0.26 to 5.61 μg·L^−1^, a lack of chronic toxicity data does not allow for a full assessment of risk, and it may present an environmental hazard [8,36]. Other benzophenonic compounds, such as benzophenone-2, also demonstrate estrogenic effects [36]. In an environmental risk assessment, 92% of the organic chemicals used for the preparation of fragrances show an acceptable environmental risk [31]. However, the design of new fragrances with properties such as better adherence to surfaces or higher stability will lead to higher persistence and lipophilicity, which may increase their risk to the aquatic environment in future [8].

## 5. Conclusions

A wide range of cleaning and PCP used in households were identified and subsequently allocated into 14 product categories. Available market research data, and comparison with usage data in the literature, gives confidence in the relative use of each product category, and the estimates on how much was used resulted in prioritization of dishwashing liquids and body-washes for evaluation of chemicals present in the products.

Assessment of the chemicals present resulted in their allocation into a range of classes, with further evaluation of surfactants, preservatives and fragrances. Surfactants are relatively well studied, and are expected to pose a low risk for the aquatic environment. However, in contrast some preservatives exhibit toxicity, including estrogenic activity, are resistant to degradation and/or bioaccumulative and may, therefore present a hazard for the aquatic environment. Additionally, fragrances include compounds exhibiting estrogenic activity, and the lack of toxicity data for these and the preservatives is of concern.

By prioritizing the product categories and classifying the chemicals, the work has demonstrated that it is possible to begin to identify gaps in knowledge and to begin to prioritize compounds, or classes of compounds, for environmental risk assessment.

## Supplementary Files

Supplementary File 1
