# Supplementary material for: An Approach for Prioritizing “Down-the-Drain” Chemicals Used in the Household
Source: Int J Environ Res Public Health. 2015 Jan 26;12(2):1351–67. doi: 10.3390/ijerph120201351 (PMC4344670; doi:10.3390/ijerph120201351)
Supplement: Supplementary File 1 [file ijerph-12-01351-s001.pdf]

An Approach for Prioritizing "Down-the-Drain" Chemicals Used in the Household

The questionnaire:

Please list up to 10 products you **most frequently** use in the bathroom and kitchen. These should be:

- Cleaning products in the kitchen (such as dishwashing liquid, dishwasher powder, fabric conditioner, disinfectant)
- Cleaning products in the bathroom (such as bleach, lime scale remover)
- Personal care products (such as shampoo, hair conditioner, toothpaste, deodorant, cream soap, soap and body cream)

Please identify the product, along with the **brand** and the **exact name** of the product. For each of these, please tick how often (daily, weekly or montly) and how much (0–10, 10–100 or >100 mL (or g)) of the product is used each day/week/month. Two examples are given below.

Product	Brand	Frequency			Quantity Used Each Day/Week/Month		
		Daily	Weekly	Monthly	0–10 mL (or g)	1–100 mL (or g)	>100 mL (or g)
Dishwashing liquid	*FAIRY clean and fresh (apple and orchard)*						
Toothpaste	*SENSODYNE Daily care*						
1.							
2.							
3.							
4.							
5.							
6.							
7.							
8.							
9.							
10.							

Table S1. The 26 different hand wash gels as reported by the respondents who used these products, in order of decreasing average use.

Brand	Full Description	Users	Frequency	Estimate of Use (mL)			Average Use
			Daily	0–10	10–100	>100	$L \cdot per^{-1} \cdot yr^{-1}$
Palmolive	Milk and Honey	4	4		4		0.83
Simple	Kind to Skin (Antibacterial)	3	3	1	2		0.62
Cien	Water Lily and Lotus	2	2		2		0.42
Dove	Beauty Cream Wash	2	2		2		0.42
Carex	(Cussons) Protect Plus (Antibacterial)	2	2	1	1		0.23
Dettol	Aloe Vera and Milk Protein	1	1		1		0.21
Dettol	Healthy Touch Moisture	1	1		1		0.21
Dove	Cucumber and Green Tea Frgrance	1	1		1		0.21
Essential Waitrose	Antibacterial	1	1		1		0.21
Faith in Nature	Tea Tree and Aloe Vera	1	1		1		0.21
Palmolive	Raspberry Heavenly Hands	1	1		1		0.21
Radox	Natural Antibacterial Butter and Ginger	1	1		1		0.21
Radox	Natural Antibacteral Pink Grapefruit and Basil	1	1		1		0.21
Sainsbury's	Original Antibacterial	1	1		1		0.21
Carex	(Cussons) Moisture Plus with vitE	1	1	1			0.02
Carex	(Cussons) Protect Plus Sensitive	1	1	1			0.02
Carex	(Cussons) Triple Protection Moisture Plus	1	1	1			0.02
Carex	(Cussons) Original	1	1	1			0.02
Champneys	Spa Treatments Citrus Blush	1	1	1			0.02
Dettol	Antibacterial Original	1	1	1			0.02
Dove	For Fine Skin	1	1	1			0.02
Lux	Peach & Cream	1	1	1			0.02
Medex	Anti-bacterial Handwash	1	1	1			0.02
Method	Waterfall Paraben Free	1	1	1			0.02
Natural Elements	Raspberry and Cranberry	1	1	1			0.02
Tesco	Natural Lime and Grapefruit Extract	1	1	1			0.02
	Total	34	34	14	20		4.63

Table S2. The 37 different shampoo products as reported by the respondents who used these products, in order of decreasing average use.

Brand	Full Description	Users	Frequency		Estimate of Use (mL)			Average Use
			Daily	Weekly	0–10	10–100	>100	$L \cdot per^{-1} \cdot yr^{-1}$
Pantene Pro-V	For Fine/Weak Hair	2	2			2		0.42
Head & Shoulders	2 in 1 Anti Dandruff Classic	5	5		4	1		0.29
Herbal Essences	Dazzling shine	4	3	1	3	1		0.25
L'Oreal	Elvive Colour protect	2	1	1		2		0.24
Alberto Balsam	Tea tree tingle	1	1			1		0.21
Cien	Provitamin anti-dandruff	1	1			1		0.21
FAST	For longer and stronger hair	1	1			1		0.21
Garnier Fructis	Colour resist	1	1			1		0.21
Herbal Essences	Beautiful ends	1	1			1		0.21
Herbal Essences	Moroccan my Shine	1	1			1		0.21
L'Oreal	Elvive Fibrology (thickening)	1	1			1		0.21
L'Oreal	ElviveNutri-Gloss	1	1			1		0.21
L'Oreal	Elvive Vitamino Colour	1	1			1		0.21
PALMOLIVE	Long and Shine	1	1			1		0.21
Syoss	Repair Therapy	1	1			1		0.21
TRESemmè	Luxurius Moisture	1	1			1		0.21
Dove	Daily care (with micro moisture serum) for Normal Hair	1	1			1		0.21
Head & Shoulders	Apple Fresh	2	2		2			0.04
L'Oreal	Elvive Antifrizz-Smooth Intense	1		1		1		0.03
L'Oreal	Elvive Triple Resist (2-in-1)	1		1		1		0.03
Palmolive	Morbidezza & Brillantezza	1		1		1		0.03
Pantene Pro-V	Repair and Rescue	1		1		1		0.03
Schwarzkopf	Professional (Ultmate Shine Superfruit)	1		1		1		0.03
Ultrex	Damaged and Coloured Hair Repair	1		1		1		0.03
VO5	Flake off 2 in 1	1		1		1		0.03
Aussie	Miracle moist	1		1		1		0.03
Fructis	Lemon (Citrus Mint)	1	1		1			0.02
Herbal Essences	Honey	1	1		1			0.02
Liz Earle	Botanic shine	1	1		1			0.02

Table S2. *Cont.*

Brand	Full Description	Users	Frequency		Estimate of Use (mL)			Average Use
			Daily	Weekly	0–10	10–100	>100	L·per^{-1}·yr^{-1}
L'Oreal	Elvive Thickening	1	1		1			0.02
OGX	Renewing Moroccan Argan	1	1		1			0.02
Syoss	Professional performance (Volume Lift)	1	1		1			0.02
Ultrex	Deep Clean Action (for men)	1	1		1			0.02
Wash & Go	Classic	1	1		1			0.02
Herbal Essences	Rose Hips, Jojoba Extracts & Vitamin E	1	1		1			0.02
Schwarzkopf	Professional (colour shine)	1		1	1			0.003
TRESemmè	Keratin smooth	1		1	1			0.003
	Total	47	35	12	20	27		4.35

Table S3. The 26 different hair conditioners as reported by the respondents who used these products, in order of decreasing average use.

Brand	Full Description	Users	Frequency			Estimate of Use (mL)			Average Use
			Daily	Weekly	Monthly	0–10	10–100	>100	$L \cdot per^{-1} \cdot yr^{-1}$
Pantene Pro-V	Normal Thick Hair	2	2				2		0.415
Herbal Essences	Beautiful Ends	2	1	1			2		0.235
Alberto Balsam	Coconut and Lychee	1	1				1		0.208
FAST	For Longer and Stronger Hair	1	1				1		0.208
FAST	For Long and Strong Hair	1	1				1		0.208
Garnier fructis	Strength and Shine	1	1				1		0.208
Herbal Essences	Moroccan my Shine	1	1				1		0.208
L'Oreal	Elvive Fibrology (Thickening)	1	1				1		0.208
L'Oreal	Elvive Full Restore 5 Extreme	1	1				1		0.208
L'Oreal	Elvive Nutri-Gloss	1	1				1		0.208
L'Oreal	Elvive Vitamino Colour	1	1				1		0.208
Palmolive	Brilliant Colour	1	1				1		0.208
Syoss	Repair Therapy	1	1				1		0.208
Dove	Daily Care (with micro moisture serum) for Normal Hair	1	1				1		0.208
L'Oreal	Elvive Antifrizz-Smooth Intense	1		1			1		0.028
Pantene Pro-V	Repair and Rescue	1		1			1		0.028
Schwarzkopf Gliss	Dry, Damaged Hair	1		1			1		0.028
TRESemmè	Cleanse and Replenish	1		1			1		0.028
Dove	Daily Shine	1	1			1			0.021
Liz Earle	Botanic Shine for Normal Hair	1	1			1			0.021
OGX	Renewing Moroccan Argan	1	1			1			0.021
Pantene Pro-V	Repair and Protect	1	1			1			0.021
Alberto Balsam	Honey and Almond	1			1		1		0.007
Schwarzkopf	Professional (Colour Protect)	1		1		1			0.003
TRESemmè	Keratin Smooth	1		1		1			0.003
Aussie	Miracle Moist	1		1		1			0.003
	Total	28	19	8	1	7	21		3.35

Table S4. The 36 different laundry products as reported by the respondents who used these products, in order of decreasing average use.

Brand	Full Description	Users	Frequency			Estimate of Use (mL)			Average Use
			Daily	Weekly	Monthly	0–10	10–100	>100	$L \cdot per^{-1} \cdot yr^{-1}$
Daz	Regular	1		1				1	0.277
Morning fresh	Non-bio (Water Lily and Jasmine)	1		1				1	0.277
Fairy	Non-bio	1	1				1		0.208
Fairy	Concentrated Fabric Softener (Sensitive)	1	1				1		0.208
Ariel	3 in 1 Biological	4		2	2		4		0.069
Lenor	Cold Orchid	1			1			1	0.069
Persil	Non-bio with Wash Booster	2		1	1			2	0.069
Essential Waitrose	Non-biological Laundry Liquid Detergent	2		2			2		0.055
Comfort	Pure (White)	2		2			2		0.055
Soupline	Grand Air	2		2			2		0.055
**Ariel**	**Febreze Excel 24 Wash**	**2**		2			2		0.055
Formil	Express	2		2			2		0.055
Surf	Lavender & Spring jasmine	2		2			2		0.055
Ariel	Actilift Colour and Style	1		1			1		0.028
Persil	Non-bio Capsules	1		1			1		0.028
Easy Breeze	Colours with Fade Resistant Formula	1		1			1		0.028
Essential Waitrose	Colour Care Laundry Liquid Detergent	1		1			1		0.028
Dixan	Original (Extra Bright)	1		1			1		0.028
Lenor	Flowers 21 (for Sensitive Skin)	1		1			1		0.028
Softlan	Apple Blossom	1		1			1		0.028
Essential Waitrose	Fresh Fabric Conditioner (concentrated)	1		1			1		0.028
Ver nel Aroma Therapy	Balsam Oil and Orchid	1		1			1		0.028
Dettol	Antibacterial (Cleanser Spring Fresh)	1		1			1		0.028
Fairy	Non-bio	1		1			1		0.028
Ariel	Brilliant Handwash and Twintub Cleaning	1		1			1		0.028
Bold	White Lily	1		1			1		0.028

Table S4. *Cont.*

Brand	Full Description	Users	Frequency			Estimate of Use (mL)			Average Use
			Daily	Weekly	Monthly	0–10	10–100	>100	$L \cdot per^{-1} \cdot yr^{-1}$
Quanto	Greek Islands	1		1			1		0.028
Persil	Small and Mighty	1		1			1		0.028
Fairy	Non-bio	1		1			1		0.028
Bold	2 in 1 Lavender	1			1		1		0.007
Ariel	Colour and Style 3-in-1 Pods	1			1		1		0.007
Bold	Lavender and Chamomile	1			1		1		0.007
Comfort	Sunshiny Days	1			1		1		0.007
Persil	Bio Capsules (Lavender)	1		1		1			0.003
Liz Earle	Cleanse and Polish	1		1		1			0.003
Surf	Herbal Extracts	1		1		1			0.003
	Total	46	2	36	8	3	38	5	1.99

Table S5. The 47 different cleaning products as reported by the respondents who used these products, in order of decreasing average use.

Product Type	Brand	Full Description	Users	Frequency			Estimate of Use (mL)			Average Use
				Daily	Weekly	Monthly	0–10	10–100	>100	$L \cdot per^{-1} \cdot yr^{-1}$
Surface cleanser	Dettol	Anti-bacterial	7	1	5	1	2	4	1	0.228
Toilet cleaner (bleach)	Duck	Germ Kill- (4-in-1)	1	1				1		0.208
General purpose cream cleaner	Cif	Classic	3		3			3		0.083
Lime scale remover	Domestos	Zero (toilet lime scale remover)	3		3			3		0.083
Bleach	Domestos	Sunfresh	2			2	1		1	0.070
Bleach	Domestos	Extended Germ Kill (Pink)	3		2	1		3		0.062
Lime scale remover	Mr Muscle	5-in-1 Shower Shine	2		2			2		0.055
All-purpose cleaner	Flash	Clean and Shine Crisp Lemons	2	1	1		1	1		0.048
Kitchen spray (for surfaces)	Flash	With Bleach	2	2			2			0.042
Lime scale remover	Viakal	Ultra Bathroom	2		2		1	1		0.030

Table S5. *Cont.*

Product Type	Brand	Full Description	Users	Frequency	Estimate of Use (mL)	Average Use	Product Type	Brand	Full Description	Users
				Daily	Weekly	Monthly				
Bathroom cleaner	Ecover	Spray	1		1			1		0.028
Bleach	ASDA	Thick Bleach (Lemon)	1		1			1		0.028
Bleach	Klinex	Chlorine Classic	1		1			1		0.028
Bleach	Sainsbury	Toilet Cleaner (thick bleach)	1		1			1		0.028
Disinfectant	Cif	Cream with Bleach (microparticles)	1		1			1		0.028
Disinfectant	SMAC	Gel with Bleach	1		1			1		0.028
Cleaning multipurpose spray	Cif	Easylift Actifizz Multipurpose Spray(Ocean)	1		1			1		0.028
Lime scale remover	CILLIT BANG	Power Spray	1		1			1		0.028
Power & Fresh Anti-Bacterial Multi-Purpose Cleaner	Dettol	Green Refreshing Apple	1		1			1		0.028
Multi-purpose cleaner cream	Asda	Lemon	1		1			1		0.028
Toilet cleaner	Flash (Febreze)	Pink Blossom	1		1			1		0.028
Toilet cleaner	Ecover	Triple Action Toilet Cleaner	1		1			1		0.028
Toilet cleaner (bleach)	Duck	Cleaner Ocean	1		1			1		0.028
Toilet cleaner	Bloo	Crystal Clean Fruity Burst	1		1			1		0.028
Bleach	Waitrose Essential	Citrus	1		1			1		0.028
Bleach	Parozone	Original	2	1	1		2			0.024
Disinfectant	Dettol	Anti-bacterial	2	1	1		2			0.024
Multi-action spray (Kitchen cleaner)	Ecover	Spray	2	1	1		2			0.024

Table S5. *Cont.*

Product Type	Brand	Full Description	Users	Frequency			Estimate of Use (mL)			Average Use
				Daily	Weekly	Monthly	0-10	10-100	>100	$L \cdot per^{-1} \cdot yr^{-1}$
Bathroom cleaner	Astonish	Spray	1	1			1			0.021
Disinfectant	Dettol	Apple	1	1			1			0.021
Kitchen spray	Tesco	24hr Anti-bacterial Ultimate	1	1			1			0.021
Lime scale remover	WC NET	Intense Ocean Fresh	1	1			1			0.021
Toilet cleaner (flash liquid)	Harpic	Pink Blossom	1	1			1			0.021
Bleach	Domestos	Original	2		1	1	1	1		0.010
Multi-purpose cleaner cream	Cif	Lemon	2		1	1	1	1		0.010
Bathroom cleanser	Dettol	Power and Pure Bathroom (with Active Oxygen)	1			1		1		0.007
Lime scale remover	Descalite	Rapid action	1			1		1		0.007
Surface cleaning cream	Tesco	Citrus	1			1		1		0.007
**Lime scale remover**	**Harpic**	**White and Shine Original**	**3**		**2**	**1**	**3**			0.006
Lime scale remover	Viakal	Spray	2		2		2			0.006
Bathroom cleaner (multi action)	Dettol	Green Apple	1		1		1			0.003
Disinfectant	Wizz	Lemon	1		1		1			0.003
Kitchen cleaning spray	Flash	Degreasing	1		1		1			0.003
Kitchen/bathroom cream cleaner	Waitrose Essential	Lemon	1		1		1			0.003
Lime scale remover	Tik Tak	Powder with Bleach	1		1		1			0.003
Multi-Purpose Cleaner	Dettol	Power & Fresh Anti-Bacterial Lemon	1		1		1			0.003
Lime scale remover	Ecover	Spray	1		1		1			0.003
	Total		72	13	49	10	32	38	2	1.57

Table S6. The 8 different dishwashing detergents as reported by the respondents who used these products, in order of decreasing average use.

Brand	Full Description	Users	Frequency		Estimate of Use (mL)			Average Use
			Daily	Monthly	0–10	10–100	>100	$L{\cdot}per^{-1}{\cdot}yr^{-1}$
Waitrose	Essential All-in-one	2	2			2		0.42
Fairy	All-in-one Lemon	1	1			1		0.21
Finish	Classic Powder	1	1			1		0.21
Finish	Quantum Regular 30S	1	1			1		0.21
W5	All-in-one	1	1			1		0.21
Finish	All-in-One Powerball	2	2		2			0.04
Tesco	Lemon	1	1		1			0.02
Finish	Power Powder Lemon	1		1		1		0.01
	Total	10	9	1	3	7		1.32

Table S7. The 31 different toothpastes as reported by the respondents who used these products, in order of decreasing average use.

Brand	Full Description	Users	Frequency	Estimate of Use (mL)			Average Use
			Daily	0–10	10–100	>100	$L{\cdot}per^{-1}{\cdot}yr^{-1}$
Colgate	Advanced White	6	6	6			0.12
Sensodyne	Daily Care	5	5	5			0.10
Colgate	Total	3	3	3			0.06
Macleans	Fresh Mint	3	3	3			0.06
Colgate	Total Advanced	2	2	2			0.04
Colgate	Max White with Whitening Stripes	2	2	2			0.04
Oral B	Pro-Expert Fluoride	2	2	2			0.04
Oral B	1.2.3 Fresh Mint Fluoride	2	2	2			0.04
Aim	Whitening	1	1	1			0.02
Aquafresh	Fresh and Minty	1	1	1			0.02
Aquafresh	Triple Protection	1	1	1			0.02
Arm and Hammer	Advanced White	1	1	1			0.02
Blanx	White Shock	1	1	1			0.02
Blend-a-Med	Classic	1	1	1			0.02
Close up	Deep Action	1	1	1			0.02
Colgate	Advanced Sensation White	1	1	1			0.02
Colgate	Sensitive Multi Protection	1	1	1			0.02
Colgate	Total Whitening	1	1	1			0.02
Colgate	Max Fresh	1	1	1			0.02
Colgate	Max White with Crystal White Mint	1	1	1			0.02
Dentalux	3 Mint Fresh	1	1	1			0.02
Oral B	Complete	1	1	1			0.02
Oral B	Pro-Expert Sensitive and Gentle Whitening	1	1	1			0.02
Sensodyne	Gentle Whitening	1	1	1			0.02

Table S7. *Cont.*

Brand	Full Description	Users	Frequency	Estimate of Use (mL)			Average Use
			Daily	0–10	10–100	>100	$L \cdot per^{-1} \cdot yr^{-1}$
Sensodyne	Pro-Expert	1	1	1			0.02
Sensodyne	Pronamel	1	1	1			0.02
Sensodyne	Pro-Schmelz	1	1	1			0.02
Sensodyne	Whitening	1	1	1			0.02
Signal	Anti-tartar Family Care	1	1	1			0.02
White Glo	Professional Choice	1	1	1			0.02
Corsodyl	Daily Original	1	1	1			0.02
	Total	48	48	48			1.00

Table S8. The 19 different deodorants as reported by the respondents who used these products, in order of decreasing average use.

Brand	Full Description	Users	Frequency	Estimate of Use (mL)			Average Use
			Daily	0–10	10–100	>100	$L \cdot per^{-1} \cdot yr^{-1}$
Dove	Invisible Dry	2	2	2			0.04
Sure	Women Cotton (Ultra Dry Roll-on)	2	2	2			0.04
Axe	Anarchy for Him (Roll-on)	1	1	1			0.02
Arrid	Antiperspirant XX for Men	1	1	1			0.02
Bionsen	Sensitive Stick	1	1	1			0.02
Dove	Cucumber and Green Tea Scent	1	1	1			0.02
Dove	Go Fresh	1	1	1			0.02
Nivea	Cool Kick Roll-on	1	1	1			0.02
Nivea	Fresh Active Men	1	1	1			0.02
Nivea	Pure Invisible Women	1	1	1			0.02
Nivea	Silver Protect Anti-bacterial Men	1	1	1			0.02
Noxzema	Roll-on Memories	1	1	1			0.02
Rock Face	48hr Antiperspirant Deodorant	1	1	1			0.02
Sanex	Dermo Extra Control	1	1	1			0.02
Sanex	Extra Effective 0%	1	1	1			0.02
Sure	Quantum Men	1	1	1			0.02
Vaseline	Active Fresh	1	1	1			0.02
Vicky	Deo-roll-on 48h Antiperspirant	1	1	1			0.02
Palmolive	Sensual Aromatherapy Jasmine and Rose Orchid Extract	1	1	1			0.02
	Total	21	21	21			0.44

Table S9. The 11 different face creams as reported by the respondents who used these products, in order of decreasing average use.

Brand	Product Description	Users	Frequency		Estimate of Use (mL)			Average Use
			Daily	Weekly	0–10	10–100	>100	$L \cdot per^{-1} \cdot yr^{-1}$
Nivea	Pure and Natural	1	1			1		0.21
Boots	Tea Tree & Witch Hazel Peel Off	1		1		1		0.03
Nivea	Daily Essentials Day Cream	1	1		1			0.02
Boots	No 7 Beautiful Skin Night Cream for Normal / Oily Skin	1	1		1			0.02
L'Oreal	Elvive Revitalift (SPF 30)	1	1		1			0.02
Eurecin	Dermo Purifier Cleanser Hydrating Care	1	1		1			0.02
Avene	Lavente Clean-AC	1	1		1			0.02
Clarins	Daily Energizer Cream (Normal to Dry Skin)	1	1		1			0.02
Simple	Kind to Skin Protecting Light Moisturising SPF 15	1	1		1			0.02
Liz Earle	Skin Repair Dry / Sensitive	1	1		1			0.02
Soltan	Moisturising Suncare Sun Lotion Water Resistant (SPF 50)	1	1		1			0.02
Total		11	10	1	9	2		0.42

Table S10. The 16 different face washes as reported by the respondents who used these products, in order of decreasing average use.

Brand	Product Description	Users	Frequency		Estimate of Use (mL)			Average Use
			Daily	Weely	0–10	10–100	>100	$L \cdot per^{-1} \cdot yr^{-1}$
Clearasil	Rapid Action Scrub (Ultra Deep Pore Treatment)	1		1		1		0.03
Garnier	Soft Essentials Comforting Toner	1		1		1		0.03
Nivea	Original for Men	1	1		1			0.02
Clarins	Gentle Foaming Cleanser with Shea Butter Dry/Sensitive Skin	1	1		1			0.02
Quinoderm	Antibacterial Facewash	1	1		1			0.02
Clinique	Skin Supplies for Men Regular Strength	1	1		1			0.02
Eau Thermale Jonzac	Cleansing Gel	1	1		1			0.02
Clean and Clear	Blackhead Clearing Cleanser	1	1		1			0.02
Clarins	Cleansing Milk Combination Oily Skin	1	1		1			0.02
Eurecin	Dermo Purifier Cleanser	1	1		1			0.02
Avene	Soapless Gel Clearance	1	1		1			0.02

Table S10. *Cont.*

Brand	Product Description	Users	Frequency		Estimate of Use (mL)			Average Use
			Daily	Weely	0–10	10–100	>100	$L \cdot per^{-1} \cdot yr^{-1}$
Simple	Kind to Skin Refreshing Facial Wash Gel	1	1		1			0.02
Neutrogena	Visible Clear (Pink Grapefruit)	1	1		1			0.02
Biotherm	Biosource Exfoliating Gel Normal/Combination Skin	1	1		1			0.02
Clinique	Anti-blemish Solution Clarifying Lotion	1	1		1			0.02
Phylosophy	Microdelivery Exfoliating Wash	1		1	1			0.003
	Total	16	13	3	14	2		0.33

Table S11. The 6 different soap bars as reported by the respondents who used these products, in order of decreasing average use.

Brand	Full Description	Users	Frequency	Estimate of Use (mL)			Average Use
			Daily	0–10	10–100	>100	$L \cdot per^{-1} \cdot yr^{-1}$
Dove	Dove Soft Peeling Gentle Exfoliating Beauty Cream Bar	5	5	5			0.10
Imperial Leather	Original	1	1	1			0.02
Waitrose	Essential Lemon Blossom and Amber	1	1	1			0.02
Lush	Pink	1	1	1			0.02
Pears	Original	1	1	1			0.02
Simple	Pure Soap	1	1	1			0.02
	Total	10	10	10			0.21

Table S12. The 7 different shaving products as reported by the respondents who used these products, in order of decreasing average use.

Brand	Full Description	Users	Frequency			Estimate of Use (mL)			Average Use
			Daily	Weekly	Monthly	0–10	10–100	>100	$L \cdot per^{-1} \cdot yr^{-1}$
Dove	Men + Care (sensitive)	1		1			1		0.028
Gillette Fu-sion	Hydra (Sensitive Shave Gel)	2	1	1		2			0.024
Lush	Fit for a prince	1	1			1			0.021
Dove	Men + Care (sensitive)	1		1		1			0.003
Nivea	Original	1		1		1			0.003
Nivea	Original	1		1		1			0.003
Cien	Sensitive	1			1	1			0.001
	Total	8	2	5	1	7	1		0.08

© 2015 by the authors; licensee MDPI, Basel, Switzerland. This article is an open access article distributed under the terms and conditions of the Creative Commons Attribution license (http://creativecommons.org/licenses/by/4.0/).
